# Effects of Curcumin Supplementation on Inflammatory Markers, Muscle Damage, and Sports Performance during Acute Physical Exercise in Sedentary Individuals

**DOI:** 10.1155/2021/9264639

**Published:** 2021-10-07

**Authors:** Kelly Aparecida Dias, Aline Rosignoli da Conceição, Lívya Alves Oliveira, Stephanie Michelin Santana Pereira, Stefany da Silva Paes, Larissa Farias Monte, Mariáurea Matias Sarandy, Rômulo Dias Novaes, Reggiani Vilela Gonçalves, Ceres Mattos Della Lucia

**Affiliations:** ^1^Department of Nutrition and Health, Universidade Federal de Viçosa, Viçosa, Minas Gerais, Brazil; ^2^Department of General Biology, Universidade Federal de Viçosa, Viçosa, Minas Gerais, Brazil; ^3^Department of Structural Biology, Universidade Federal de Alfenas, Alfenas, Minas Gerais, Brazil

## Abstract

Exhaustive and acute unusual physical exercise leads to muscle damage. Curcumin has been widely studied due to the variety of its biological activities, attributed to its antioxidant and anti-inflammatory properties. Furthermore, it has shown positive effects on physical exercise practitioners. However, there is no literature consensus on the beneficial effects of curcumin in acute physical activities performed by sedentary individuals. Therefore, we systematically reviewed evidence from clinical trials on the main effects of curcumin supplementation on inflammatory markers, sports performance, and muscle damage during acute physical exercises in these individuals. We searched PubMed/MEDLINE, Scopus, Web of Science, and Embase databases, and only original studies were analyzed according to the PRISMA guidelines. The included studies were limited to supplementation of curcumin during acute exercise. A total of 5 studies were selected. Methodological quality assessments were examined using the SYRCLE's risk-of-bias tool. Most studies have shown positive effects of curcumin supplementation in sedentary individuals undergoing acute physical exercise. Overall, participants supplemented with curcumin showed less muscle damage, reduced inflammation, and better muscle performance. The studies showed heterogeneous data and exhibited methodological limitations; therefore, further research is necessary to ensure curcumin supplementation benefits during acute and high-intensity physical exercises. Additionally, mechanistic and highly controlled studies are required to improve the quality of the evidence and to elucidate other possible mechanisms. This study is registered with Prospero number CRD42021262718.

## 1. Introduction

Exhaustive physical exercise, especially acute or high intensity with many eccentric contractions, leads to muscle damage and delayed onset muscle soreness (DOMS) [1–3]. The disruption of the sarcolemma can characterize muscle damage, in addition to cytoskeletal damage, distortion of contractile components, and extracellular abnormalities of the myofibril matrix [4]. Furthermore, exercise-induced muscle damage (EIMD) induces an inflammatory response associated with decreased muscle strength, decreased ROM, localized swelling, and an increase in muscle proteins in the blood, such as creatine kinase (CK) [5]. It also raises markers such as C-reactive protein (CRP) and inflammatory interleukins and contributes to the production of reactive oxygen species (ROS) by promoting the activation of transcription factors, such as the nuclear factor nuclear-*κ*B (NF-*κ*B) [6–8].

Impaired muscle function caused by EIMD and DOMS and subsequent inflammatory responses can affect sports performance. In this sense, strategies capable of controlling or minimizing muscle damage and exacerbated inflammatory responses have been increasingly studied. In this regard, nutritional supplements with antioxidant and anti-inflammatory properties have represented an alternative for such purposes [9, 10]. Among the alternatives, curcumin (1,7-bis (4-hydroxy-3-methoxyphenyl) 1,6-heptadiene-3,5-dione) stands out, being the main polyphenol of *Curcuma longa* L. [11, 12]. The Food and Drug Administration (FDA) of the United States has listed curcumin as “Generally Recognized as Safe” (GRAS), and supplements containing curcumin have been approved for human consumption [13].

A recent review has shown that curcumin has various biological activities, thanks to its antioxidant and anti-inflammatory properties, which could be cardioprotective, immune-regulating, antineoplastic, and hepatoprotective effects, in addition to positive effects on diabetes and the nervous system [14]. Moreover, it has shown positive effects on exercise practitioners and athletes. A clinical trial with individuals of both sexes has shown that, after eccentric exercise, supplementation with curcumin (500 mg) significantly reduced EIMD and CK concentrations, leading to better recovery after exercise [15]. Another study has also shown that curcumin significantly decreased CK levels and muscle pain in men undergoing muscle damage protocol [16]. The study by Sahin et al. [17] showed that curcumin prevented muscle damage and improved performance in animals by regulating the pathways of NF-*κ*B and nuclear factor derived from erythroid 2-like 2 (Nrf2).

From what is known, although curcumin has been extensively studied due to its beneficial potential in physical exercise and sports performance, there is no literature consensus on its effects on acute and/or high-intensity physical exercises. Therefore, from clinical trials, we have summarized the available evidence of the effects of curcumin supplementation on inflammatory markers, sports performance, and muscle damage during acute physical exercises in sedentary individuals.

## 2. Methods

### 2.1. Protocol and Registration

This systematic review was guided by the following research question: “What is the impact of curcumin supplementation on sports performance in sedentary individuals? Second, what are the main inflammatory mechanisms involved in this process? Third, what are the main methodological parameters used to assess the effect of curcumin supplementation on sports performance?” This systematic review was developed according to the Preferred Reporting Items for Systematic Reviews and Meta-Analyses (PRISMA) guidelines [18].

### 2.2. Eligibility Criteria

This study included randomized-controlled clinical trials with sedentary individuals older than 18 years submitted to acute physical exercise. In contrast, studies carried out with children, teenagers, seniors, and physically active individuals were excluded. Concerning the intervention type, we have included studies with any dosage and form of curcumin supplementation. Studies in which curcumin supplementation has occurred in combination with other foods or medication were excluded.

### 2.3. Search

This systematic review was developed according to the Preferred Reporting Items for Systematic Reviews and Meta-Analysis (PRISMA) guidelines [18], which were used to guide the selection, screening, and eligibility of studies. The participants, intervention comparators, outcomes, and study design (PICOS) criteria adopted in this study are shown in Table 1.

Six authors (ARC, KAD, LFM, LAO, SMSP, and SSP) independently searched for original articles using the following electronic databases: PubMed/MEDLINE (https://www.ncbi.nlm.nih.gov/pubmed), Scopus (https://www.scopus.com/home.uri), Web of Science (https://www.webofknowledge.com), and Embase (https://www.embase.com). The descriptors were structured based on search filters built for three domains: (i) curcumin, (ii) exercise, and (iii) human. The PubMed/MEDLINE platform filters were constructed using a hierarchical distribution of the MeSH terms (Medical Subject Headings) and by the algorithm TIAB (Title and Abstract). These filters were adapted for research in the Scopus platform, Web of Science, and Embase; however, the filter for the original article was provided by the Scopus platform (Table S1). The search strategies were not limited by date and language. The bibliographic search was performed on June 28, 2021.

### 2.4. Study Selection

The authors (ARC, KAD, LFM, LAO, SMSP, and SSP) selected eligible studies following the analysis of their titles and abstracts. The level of agreement among these reviewers was assessed using kappa (kappa = 0.909). The information was extracted independently and analyzed separately. After reading titles, abstracts, and full-text analysis, we have included all randomized controlled trials that assessed the effects of curcumin consumption in sedentary adults submitted to acute physical exercise. Letters, reviews, observational studies, book chapters, abstracts, unpublished articles, *in vitro* studies, animal experiments, studies with physically active individuals, and studies that associated curcumin supplementation with another food or medicine were excluded. In addition, two reviewers (KAD and LAO) manually searched the reference lists of the studies selected in the previous step independently evaluated to find additional relevant articles. Selections were then compared, and differences were resolved in consultation with another three reviewers (MMS, RVG, and CMDL).

### 2.5. Data Extraction Process

After selecting the clinical trials, the data of the publications were extracted using standardized information such as an author's name, year of publication, the country where the study was held, sample characteristics (sample size, average age, and anthropometric data), study design, follow-up, intervention characteristics (dose and form of consumption), and primary and secondary outcomes. After the data extraction step, the researchers compared the data to ensure integrity and reliability.

### 2.6. Risk of Bias

The quality and risk of bias in the studies' methodology were assessed by the criteria described on the SYRCLE's risk-of-bias (RoB) tool (Systematic Review Centre for Laboratory animal Experimentation) [19]. The following methodological domains based on RoB were evaluated according to five domains of bias: (1) selection bias (random sequence generation, baseline characteristics, and allocation concealment), (2) performance bias (random housing and blinding of caregivers and/or investigators), (3) detection bias (random outcome assessment, blinding of outcome assessment), (4) attrition bias (incomplete outcome data), (5) reporting bias (selective outcome reporting), and other bias (the ethics committee and statistics).

The studies were categorized independently by the authors in three levels of bias according to the items in the RoB tool and were scored as “low risk of bias,” “high risk of bias,” or “unclear” (when the experimental information was not sufficient for categorization). We constructed a figure in the Review Manager® 5.4 program from Cochrane Collaboration (RoB 2.0) to demonstrate the risk of bias across all included studies (Copenhagen: The Nordic Cochrane Center 2020).

### 2.7. Data Analysis

All the clinical trials reviewed in this article were summarized in a standard data extraction model, according to the main characteristics and results of sports performance, muscle damage, and inflammation markers (Table 2). The studies were chronologically ordered by year of publication. Inflammatory and muscle performance markers were considered the primary outcomes. In addition, we analyzed the effect of the tested dose concerning the duration of clinical trials.

## 3. Results

### 3.1. Study Selection

**Figure 1:**
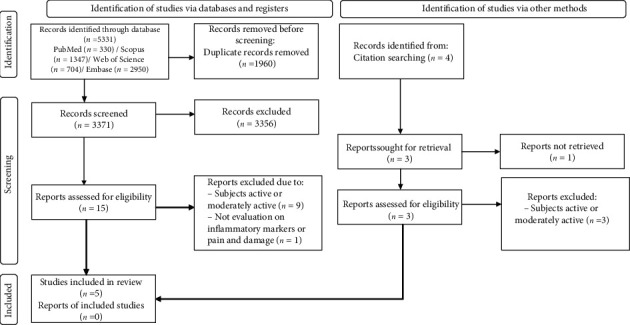
PRISMA diagram. Different phases of the selection of studies for conducting qualitative and quantitative analyses. Flow diagram of the systematic review literature search results. Based on [18].

The flowchart with the number of selected and excluded articles in each stage was built according to the PRISMA guidelines (Figure 1). After searching PubMed, Scopus, Web of Science, and Embase, we have identified 5331 articles. Posteriorly, 1960 duplicates were removed, resulting in 3371 articles, of which 3356 were excluded after reading the titles. After reading the abstracts and full text, we have excluded ten articles. Nine evaluated the effect of curcumin consumption on physically active or moderately individuals, and one did not evaluate parameters of interest, such as inflammatory markers or pain and damage muscle. We have performed the citation search to identify other relevant studies; however, none met the eligibility criteria. Finally, five articles were included in this study.

### 3.2. Description of Included Studies

In the five studies included in this review, 98 young adult individuals of both sexes, sedentary and with no comorbidities, were studied. The sample size ranged from 12 to 28 individuals. As for the study sites, three of them were conducted in Japan [2, 3, 20], one in China [21], and one in the United States [22]. All studies were randomized and controlled. The intervention time ranged from 2 days to 4 weeks.

The type of acute exercise performed varied. Regarding the type of acute exercise performed, it was observed that the majority (*n* = 3; 60%) performed eccentric contractions of the elbow flexors, one study (20%) performed a test of six sets of ten leg press repetitions, and one study (20%) performed a three-rep set of drop jump high (DJH) 70%, DJH 100% (defined as the highest jump height), and DJH 130% and an acute bike challenge. In most studies (*n* = 4; 80%), the curcumin was offered as capsules and in one (*n* = 20%) of them in the form of nanobubble water extract. In all studies (100%), curcumin was ingested alone, and the control group was curcumin-free.

One study (20%) evaluated the effect of consumption of curcumin, divided into two doses, one before exercise and the other 12 hours after exercise, and one study (20%) used curcumin for six days, starting two days before performing acute physical exercise and continuing three days after. Other two studies (40%) also offered curcumin before and after eccentric exercise; in this way, one study evaluated the supplementation of curcumin for seven days before the performance of eccentric exercise of the elbow flexors (PRE group) and for four days after the exercise (POST group); other study evaluated this supplementation in two experimental groups: one group received curcumin for seven days before acute exercise and the other for seven days after exercise. Lastly, one study (20%) evaluated the use of curcumin on performance in acute exercise at two time points, presupplementation and after four weeks (postsupplementation).

The amount of curcumin varied from 180 to 400 mg per day. Two studies (40%) evaluated the effect of 180 mg/day of curcumin supplementation for up to 7 days. One study (20%) offered 230.9 mg/day of curcumin for four weeks. Another study (20%) evaluated the single use of 300 mg/day of curcumin divided into two doses of 150 mg. In addition, one study (20%) evaluated the use of 400 mg/day of curcumin for up to 6 days (Table 3).

### 3.3. Studies' Main Results

#### 3.3.1. Inflammation

Most studies included in this review (*n* = 3; 60%) evaluated inflammatory markers. Of these, three studies (60%) evaluated the proinflammatory cytokine such as tumor necrosis factor-*α* (TNF-*α*), which regulates multiple biological processes and is a mediator of inflammation. One study showed a reduction of TNF-*α*. Two studies (*n* = 40%) also evaluated the proinflammatory cytokines interleukin-6 (IL-6) and interleukin-8 (IL-8). Decreased expression of these cytokines was observed only for IL-6 and IL-8 in 1 and 2 days after curcumin exposure. These interleukins are related to the acute inflammatory response, stimulating the release of acute-phase proteins, as well as neutrophil chemotaxis [23]. This acute phase, mediated by proinflammatory cytokines, is generally followed by the expression of anti-inflammatory cytokines (Wang et al., 2016). Only one study (*n* = 20%) evaluated the anti-inflammatory cytokine interleukin-10 (IL-10); however, there was no difference in these cytokines between treated and receiving placebo in 6 days of treatment.

McFarlin et al. found in a study with 28 individuals that supplementation of 400 mg/day of curcumin significantly reduced the levels of IL-8 (-21%; *p* = 0.030) at 1 day (-21%) and 2 days (-18%) and TNF-*α* (-25%; *p* = 0.028) at 1 day (-25%), 2 days (-23%), and 4 days (-23%) after muscle damage induced by acute leg press exercise. Despite the lack of significance, IL-6 demonstrated a similar response to IL-8 and TNF-*α* for the curcumin condition [22].

In another study with 20 healthy men, Tanabe et al. found that IL-8 serum concentration 12 h after the resistance exercise was lower (*p* = 0.033) for the supplementation of 180 mg/day of curcumin (experiment 1). However, TNF-*α* concentration remained similar between groups [3]. Thus, the curcumin supplementation significantly reduced inflammation resulting from acute physical exercise (Table 2).

#### 3.3.2. Muscle Pain and Damage

All studies evaluated creatine kinase (CK) (*n* = 5; 100%) as an essential clinical biomarker for muscle damage. Thus, most studies (*n* = 3; 60%) observed that treatment with curcumin could reduce CK activity, showing the benefits of curcumin supplementation in reducing pain and muscle damage by decreasing CK. Serum CK is an important clinical biomarker for muscle damage, such as muscular dystrophy, severe muscle destruction, and a marker of peripheral fatigue during physical exercises [21, 22]. In addition, most studies (*n* = 4; 80%) evaluated muscle pain; two of these (50%) reported a reduction in muscle pain. Only one study (20%) evaluated skeletal muscle injury and observed a decrease in the index of a knee injury.

Although inconclusive, studies have shown that curcumin was effective for some aspects of muscle damage and pain after the performance of acute physical exercises. Tanabe et al. found in a crossover study with 14 healthy young men that the consumption of a single dose of 150 mg of curcumin 1 hour before and another dose of 150 mg 12 hours after 50 maximum eccentric contractions of the elbow flexors reduced the peak of CK activity, compared to the placebo condition (peak: 7684 ± 8959 IU/L vs. 3398 ± 3562 IU/L; *p* < 0.05).

Similar findings were reported by McFarlin et al. when they found that curcumin resulted in a blunted CK response (*p* = 0.035) at 1 day (-44%), 2 days (-49%), 3 days (-57%), and 4 days (-69%) following performing the acute exercise.

In addition, Tanabe et al. [3] also found a reduction in CK concentrations (*p* = 0.020) 5-7 days after exercise and a reduction in the score for muscle pain for the group supplemented with curcumin (experiment 2) 3–6 days after the exercise when compared to the placebo group. In turn, Tanabe et al. [20] observed that the group supplemented with 180 mg/day of curcumin for 4 days presented reduced muscle pain by palpation on the 3rd day postresistance exercise and reduced muscle pain by the extension of the elbow joint when compared the placebo group.

Finally, Wang et al. found in a study that the supplementation of 230.9 mg/day of the nanobubble water curcumin extract (NCE) before performing a series of drop jumps significantly reduced the knee injury index (15.38%; *p* = 0.0467) (peak vertical ground reaction force (PVGRF)); these results showed that NCE improved skeletal muscle injury [21].

#### 3.3.3. Muscle Performance and Fatigue

Supplementation with curcumin has shown improvement in some markers of muscle performance during acute eccentric exercise. Most studies (*n* = 3; 60%) evaluated a range of motion (ROM) and maximal voluntary contraction (MVC) torque as markers of muscle performance and recovery. Two studies (40%) reported improvement in a range of motion (ROM); also, curcumin supplementation improved MVC torque in two studies (40%), which suggests that supplementation contributed to muscle recovery and performance. To reflect better performance during exercise, fatigue needs to be reduced. Lactate is an oxidizable substrate in skeletal muscle and a precursor to gluconeogenesis in muscles after exercise [24]. In addition, peripheral and central fatigue levels are related to increased ammonia levels during exercise [21]. Only one study (20%) evaluated muscle strength and muscle fatigue markers (lactate and ammonia (NH_3_), reporting increased muscle strength through increased contact time in drop jumps and reduction of lactate and NH_3_ by supplementation with curcumin.

The supplementation of 180 mg/day of curcumin improved ROM (3–4 days following the exercise) compared to the placebo group, indicating an improvement in sports performance [20]. In addition, the magnitude of decrease in MVC torque for the curcumin condition was significantly smaller immediately after exercise and at 48–96 h after exercise by 13–16% compared to the placebo group [2].

In support of these findings, Tanabe et al. observed that supplementation of 180 mg/day of curcumin (for 7 days—after acute exercise) showed improvement in MVC torque and ROM compared to the placebo group [3]. Another study [21] has found that the contact time in drop jump high was significantly increased by supplementation with NCE (230.9 mg/day for 4 weeks) (*p* = 0.0487). This increased contact time was due to the increased muscle strength due to the NCE supplementation, corroborating the statement of curcumin's role in improving sports and muscle performance. They also have reported a reduction in lactate (18.67%; *p* = 0.0057) and NH_3_ levels (9.02%; *p* = 0.0048) for the group supplemented with curcumin compared to the placebo group, indicating a contribution to reducing fatigue and improving performance.

#### 3.3.4. Biochemical and Oxidative Markers

Only one study (20%) evaluated the effects of curcumin supplementation regarding biochemical markers. Biochemical analyses can provide clinical information about the subject's physiological adaptation. NCE supplementation showed functional activities related to physiological protection and promotion of recovery [21]. Wang et al. found that curcumin consumption significantly decreased hepatic alanine aminotransferase (ALT) (35.66%) and aspartate aminotransferase (ALP) (28.65%) levels compared to the placebo condition. As for lipid-related parameters, triglycerides (TG) were significantly reduced (31.96%) in the treated group compared to the placebo group after four weeks of supplementation. In addition, high-density lipoprotein (HDL) is increased by 1.17 times compared to the placebo group [21].

Regarding oxidative markers, a single study (20%) assessed the levels of serum concentration of derivatives of reactive oxygen metabolites (d-ROMs) and the biological antioxidant potential (BAP). However, there were no significant differences between curcumin and placebo conditions [3].

#### 3.3.5. Risk of Bias

**Figure 2:**
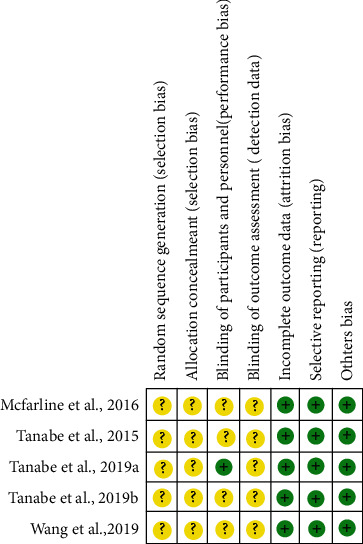
Risk of bias summary: review authors' judgments about the risk of bias item for each included study. The items in the Systematic Review Centre for Laboratory animal Experimentation (SYRCLE) risk of bias assessment were scored with “yes” indicating low risk of bias, “no” indicating high risk of bias, or “unclear” indicating that the item was not reported, resulting in an unknown risk of bias. Green: low risk of bias; yellow: unclear risk of bias; red: high risk of bias.

Regarding the selection bias of the participants, the five articles (*n* = 5; 100%) included in this review were randomized; however, they reported insufficient data on the process of sequence generation and allocation secrecy, making evaluation difficult. As for the blinding of participants and personnel, most of the studies (*n* = 4; 80%) were double-blind, thus displaying a low risk for this bias. All studies (*n* = 5; 100%) had a low risk of bias concerning data recording on incomplete outcomes since the missing data were balanced among the groups. In terms of the selected outcome reporting, all studies (*n* = 5; 100%) were classified as low risk of bias since the study protocol is not available, but the published articles include all expected results (Figure 2). All studies followed the methodology and presented the proposed results and approval on the ethics committee and information regarding the statistics used.

## 4. Discussion

**Figure 3:**
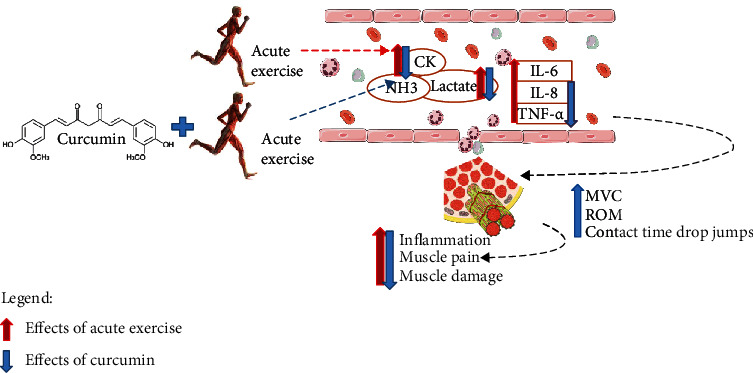
Potential positive effects of curcumin supplementation during acute physical exercises in sedentary individuals. CK: creatine kinase; NH3: ammonia; IL-8: interleukin-6; IL-8: interleukin-8; TNF-*α*: tumor necrosis factor-alpha; MVC torque: maximum voluntary contraction torque; ROM: range of motion.

Our study performed a systematic review to investigate the available scientific evidence of the effects of curcumin supplementation on inflammatory markers, muscle damage, and sports performance during acute physical exercise in sedentary individuals. In addition, we analyzed the methodological quality of studies that address this issue. We hope that these guidelines can improve research quality, reproducibility, and viability and yield studies with a low risk of bias, especially about the food supplementation in inflammatory diseases. In this sense, our review showed that curcumin supplementation reduced inflammation and muscle pain resulting from acute physical activity. In addition, it has improved muscle recovery and sports performance and reduced fatigue (Figure 3).

Regular physical exercise promotes health benefits [25]. However, long-term acute and intense physical exercise, associated with the insufficient recovery period, results in muscle damage, increased reactive oxygen species (ROS), and inflammation [26, 27]. The production of ROS can alter cellular functions and cause inflammation, leading to increased fatigue, decreased muscle function, and performance [28–30]. When practiced with adequate intensity and volume, postexercise inflammatory responses are physiological and indispensable for regenerating damaged muscles. However, they can impair muscle regeneration when uncontrolled, leading to oxidative damage, protein catabolism, and late-onset muscle pain, resulting in decreased sports performance [31, 32]. In this sense, controlling or minimizing marked inflammatory responses and muscle damage can promote faster recovery, maximize training and performance, and prevent injuries [2].

Thus, supplementation with dietary compounds has been increasingly frequent for potential use in improving sports performance and accelerating postexercise recovery. Scientific evidence has shown curcumin's potential in reducing postexercise inflammation [17, 20, 22]. The mechanisms involved are related to its ability to modulate proinflammatory cytokines and signaling pathways, in addition to its effectiveness in blocking the increased activation of NF-*κ*B, which in turn regulates the expression of TNF-*α* and inflammatory proteins [17, 33–37].

The proinflammatory cytokines TNF-*α*, IL-6, and IL-8 were evaluated as markers of inflammation by the studies included in this systematic review [2, 3, 22]. Tanabe et al. [2] observed no effect of curcumin supplementation for TNF-*α*. However, McFarlin et al. [22] reported sustained suppression of TNF-*α* in the curcumin-supplemented group compared to the placebo group. Curcumin's effectiveness in reducing TNF-*α* may be subject to a minimum dosage of 400 mg administered before and for 72 hours after exercise, which may explain the differences observed concerning the studies by Tanabe et al. [2].

McFarlin et al. [22] reported that supplementation with 400 mg of curcumin effectively reduced IL-8 levels resulting from exercise practice. In the study by Tanabe et al. [3], 7 days of 180 mg of curcumin supplementation before exercise also reduced the serum concentration of IL-8 12 hours after exercise. McFarlin et al. [22] observed that the inhibition of the transcription factor NF-*κ*B could be related to the reduction of IL-8 levels and the tendency to decrease to IL-6. Studies have shown that the promoters of the cytokines IL-6 and IL-8 have binding sites for NF-*κ*B, C/EBP*β*, and c-Jun [38, 39]. Thus, curcumin's therapeutic potential in inflammation may be related to its direct action of inhibiting NF-*κ*B, which influences the regulation of the expression of IL-6 and IL-8 [40].

Recent studies with protocols that used curcumin supplementation ranging from 0.18 g to 0.4 g/day also have shown benefits in reducing pain and muscle damage, which reflects in the decrease in serum CK [2, 3, 22]. Intense exercise leads to an increase in muscle injury markers, such as CK and lactate dehydrogenase (LDH), which are used as an indication of increased muscle damage induced by exercise (EIMD) [41, 42]. EIMD can affect muscle pain performance and enzyme activity, reducing the sports performance of athletes and physical activity practitioners [42–44].

In this sense, all studies included in this review evaluated the ability of curcumin to mitigate muscle pain and damage, in addition to preventing injuries [2, 3, 20–22]. In two studies, Tanabe et al. [3, 20] described that the administration of 180 mg of curcumin (Theracurmin-Theravalues®) caused a significant reduction in muscle pain when administered four [3] and seven [20] days later the eccentric contraction exercise.

However, Tanabe et al. [2] have found no significant effect on muscle pain using 150 mg of curcumin or placebo orally before and 12 hours after eccentric exercise. In addition, Mcfarlin et al. [22] have also reported no difference in subjective quadriceps muscle pain or daily life activities when investigating the effect of supplementation with curcumin (400 mg 24 hours before and 72 hours after) compared to placebo. Thus, curcumin supplementation may have affected pain and muscle damage when administered on recovery days [3] and for a more extended period [20]. Even so, differences in muscle pain results may be due to the subjective and individual perception of pain intensity among participants of the studies.

The effect of curcumin supplementation on serum CK activity levels after exercise was investigated by four studies included in this review [2, 3, 20, 22]. Three of them have shown significantly lower serum CK levels in the group supplemented with curcumin when compared to the placebo group [2, 3, 22]. Although Tanabe et al. [20] did not report significant differences, they observed that CK levels showed less tendency to increase in the group supplemented with curcumin.

Thus, it is possible that the direct action of curcumin in the inhibition of NF-*κ*B can inhibit enzymes that generate ROS, such as COX-2 [39]. It has also been reported that another possible mechanism for reducing CK activity through curcumin is the inhibition of the production of histamine and prostaglandin due to the suppression of the positive regulation of COX-2, showing a protective effect of the membrane, influencing vascular permeability. This inhibition reduces the permeability of the membranes, reducing the intracellular flow of CK. Thus, vascular permeability may be the key factor in reducing muscle inflammation and pain caused by EIMD [45, 46]. Although the mechanisms linked to the positive effects of curcumin during physical activities, as well as the doses and exposure time, are poorly understood, the evidence on the anti-inflammatory potential of these molecules is more objective and suggests a reduction in proinflammatory markers promoting a rapid tissue recovery after stress.

The EIMD triggers an inflammatory response associated with decreased muscle strength, ROM, localized swelling, DOMS, and increased CK, lactate dehydrogenase (LDH), and myoglobin (Mb) [5]. Mechanical stress during acute exercise and subsequent inflammatory responses lead to reduced muscle performance. Thus, MVC and ROM changes reflect the EIMD dimension, and therefore, these parameters can be used as performance markers [47]. In this sense, Tanabe et al. [2] and Tanabe et al. [20] demonstrated curcumin's action on muscle performance. They have found that curcumin supplementation 180 mg/day for 4 and 7 days after performing eccentric exercises, respectively, improved the range of motion (ROM). The same group of researchers has found the improvement in MVC torque or the lesser magnitude of its decline among those who consumed curcumin after exercise [2, 3].

The MVC and ROM markers are decreased by the activation of NF-*κ*B under high mechanical stress caused by the excessive use of some joints in sport, which generates fragments of the extracellular matrix of bone or cartilage [47]. Innate immunity recognizes these fragments by toll-like receptors. The cellular activation of NF-*κ*B mediated by this process stimulates the secretion of products responsible for the production of tissue damage, such as inflammatory cytokines (IL-1, IL-1b, IL-2, IL-15, IL-21, and TNF-*α*), chemokines (CCL-19, CCR-7), and metalloproteases (MMP-13, ADAMSTS-4) (Wainstein GE 2014). The curcumin action probably minimizes the tissue's disabling effects, blocking the NF-*κ*B signaling pathway and thus providing less muscle damage and a consequently smaller decrease in MVC and ROM [48, 49].

In addition, the increased serum concentrations of NH_3_ and lactate during exercise are related to muscle fatigue and intensify according to the exercise's intensity and length. High concentrations of lactate and ammonia indicate an increased concentration of hydrogen ions and consequent muscle acidosis, which reduces muscles' strength and working capacity [50]. Wang et al. [21] reported a reduction in lactate and ammonia (NH3) levels among participants who ingested curcumin compared to the placebo group, consequently showing a reduction in muscle fatigue.

The results by Wang et al. [21] also show that consumption of 230.9 mg/day of curcumin before acute exercise decreased exercise-induced liver injury indicators (aspartate aminotransferase (AST) and alanine aminotransferase (ALT)) and improved lipid metabolism through decreased triglycerides (TG) and increased levels of high-density lipoprotein (HDL) compared to the placebo group. In turn, ROS is related to the disruption of the cell membrane integrity caused by lipid peroxidation, with the consequent release of specific cytosol enzymes or proteins, such as AST and ALT in the blood [51].

Curcumin supplementation reduces damage and improves liver inflammation [52] by inhibiting NF-*κ*B activation, decreasing ICAM-1, COX-2, and MCP-1, and reducing intrahepatic gene expression of monocyte chemoattracting protein-1, CD11b, procollagen type I, and tissue metalloproteinase inhibitor [53, 54]. In addition, this compound can affect adiposity and lipid metabolism through several mechanisms, including the modulation of energy metabolism and inflammation [55]. In clinical practice, there is evidence about the effectiveness of curcumin supplementation in reducing the levels of plasma triglycerides and cholesterol [36, 56, 57], which suggests its hypocholesterolemic effect [17].

Curcumin has been approved by the Food and Drug Administration (FDA) as a safe compound [13]. In addition, clinical trials have assessed its safety and concluded that dosages up to 12 g/day are safe and nontoxic for human consumption for three months [13, 58]. Only one [22] out of the five studies included in this review evaluated the side effects of curcumin supplementation, and no adverse gastrointestinal effects were reported. Despite the positive reports described in this review, we believe it is necessary to evaluate the results with caution due to heterogeneity of the individual studies, mainly concerning methodological consistency to interpret the evidence available and so to avoid reporting bias and consequently to decrease the reproduction of the error through the research process.

## 5. Strength and Limitations

Considering a more comprehensive analysis of the scientific evidence in clinical trials, the risk of bias was assessed as a quality criterion complementary to the information reported in the studies reviewed. The main methodological limitations were identified in all studies analyzed. Surprisingly, no study fulfilled all methodological criteria; this finding indicates that specific reporting bias has been systematically reproduced through the research process, despite advances in regulatory strategies to stimulate the development of robust clinical research. Bias analysis showed that key characteristics such as random sequence generation or random outcome assessment and blinding of participants (caregivers and outcome evaluator) were not reported with sufficient information in the studies. Due to the marked variability identified in the reviewed studies (i.e., characteristics related to population, treatment protocols, and outcome evaluation), the findings of this systematic review were qualitatively presented. Thus, it was not possible to evaluate the results based on a meta-analysis [59, 60]. In addition, the funnel plot method was not used to explore heterogeneity since the number of included studies was less than the minimum recommended (10) for this type of analysis [61]. We believe that the results presented in this study are important and valuable as they bring new knowledge about the potential use of curcumin and its benefits in the practice of strenuous exercise by sedentary individuals. However, they should also be treated with caution, as the low number of clinical trials found on the subject can be pointed out as a limitation of this review because when there are fewer studies, the power of the tests is too low to distinguish chance from real asymmetry [61–63]. In this context, further studies are needed to ensure the benefits of curcumin supplementation during acute and high-intensity physical exercise and to elucidate other possible mechanisms.

## 6. Conclusion

Curcumin supplementation has been shown to improve sports performance, providing less EIMD and reducing fatigue by decreasing CK activity. In addition, curcumin exerts an anti-inflammatory effect by modulating proinflammatory cytokines. Curcumin supplementation is safe and probably represents beneficial sport potential, demonstrating effectiveness before and/or after acute physical exercise in sedentary individuals.

## Figures and Tables

**Table 1:** PICOS criteria for study inclusion.

Parameter	Inclusion criteria
Participants	Physically inactive adults
Intervention or exposure	Consumption of curcumin Acute physical exercise
Comparison	Placebo (no curcumin supplementation)
Outcome	Inflammation markers, muscle pain, and damage, sports performance and fatigue, biochemical and oxidative markers
Study design	Randomized clinical trials

**Table 2:** Main characteristics of the studies included in the systematic review.

Author, year, country	Sample characteristics	Study design	Intervention characteristics	Follow-up	Exercise test	Main results (curcumin *vs.* control groups)	
						Primary outcome	Secondary outcome
Tanabe et al. (2015) Japan	*n*: 14 young individuals Gender: M: 14 Age: 23.5 ± 2.3 years old Weight: 65.2 ± 11.3 kg	Single-blind crossover randomized controlled trial	G1: 300 mg/day of curcumin (Theracurmin-Theravalues®) G2: 300 mg/day of placebo (starch)	1 day Capsules administered 1 h before (150 mg) and 12 h after exercise (150 mg)	50 maximal eccentric contractions of the elbow flexors separated by 4 weeks	↔ IL-6, TNF-*α* ↓ CK ↔ muscle pain ↔ ROM ↑ MVC torque	↔ Total work, mean peak torque, upper-arm circumference
McFarlin et al. (2016) USA	*n*: 28 young individuals Gender: M: 10 W: 18 Age: G1: 20 ± 1 years old G2: 19 years old Weight: G1: 62.4 ± 11.4 G2: 65.0 ± 10.3	Double-blind randomized controlled trial	G1: 400 mg/day of curcumin (long-life) G2: 400 mg/day of placebo (rice flour)	6 days 48 h before exercise and for 72 h after	6 series of 10 repetitions of eccentric exercise (leg press) with initial load set at 110% of the estimated 1RM	↓ IL-8 and TNF-*α* ↔ IL-6 and IL-10 ↓ CK ↔ muscle pain	↔ ADL
Tanabe et al. (2019a) Japan	*n*: 24 healthy adults Gender: M: 24 Age: PRE: 28.8 ± 3.6 years old POST: 29.8 ± 3.4 years old CON: 28 ± 3.2 years old Weight: PRE: 65.2 ± 11 kg POST: 71.2 ± 5.6 kg CON: 65.7 ± 5.9 kg	Single-blind parallel randomized trial	PRE: 180 mg/day of curcumin capsules (Theracurmin-Theravalues®) POST: 180 mg/day of curcumin (Theracurcumin-Theravalues®) CON: 180 mg/day of placebo	PRE: 7 days before exercise POST: 4 days after exercise CON: 4 days after exercise	30 eccentric contractions of the elbow flexors	↔ CK (PRE and POST) ↓ muscle pain on POST ↑ ROM on POST ↔ MVC torque, ROM initially	↔ total work during exercise
Tanabe et al. (2019b) Japan	*n*: 20 healthy adults Gender: M: 20 Age: Exp 1: 28.5 ± 3.4 years old Exp 2: 29 ± 3.9 years old Weight: Exp 1: 64.9 ± 10.1 kg Exp 2: 70.7 ± 5.8 kg	Double-blind crossover, randomized controlled trial	Exp 1: G1: 180 mg/day of curcumin capsules (Theracurmin-Theravalues®) G2: placebo (starch) Exp 2: G1: 180 mg/day of curcumin capsules (Theracurmin-Theravalues®) G2: placebo (starch)	Exp 1: 7 days before exercise Exp 2: 7 days after exercise	30 eccentric contractions of the elbow flexors	Exp 1: ↓ IL-8 ↔ TNF-*α* ↔ CK, muscle pain ↔ MVC, ROM Exp 2: ↔ IL-8, TNF-*α* ↓ CK, muscle pain ↑ MVC torque, ROM	Exp 1: ↔ total work during exercise, d-ROMs, T_2_, BAP Exp 2: ↔ total work during exercise, d-ROMs, T_2_, BAP
Wang et al. (2019) China	*n*: 12 healthy young individuals Gender: W: 12 Age: 21.2 ± 1.1 years old Weight: G1: 57.1 ± 5.1 kg G2: 56.5 ± 7.6 kg	Double-blind, parallel, randomized, controlled trial	G1: 230.9 mg/day of NCE G2: 15 g/day placebo (maltodextrin)	4 weeks	A single test of 3 repetitions of drop jumps high (DJH) 70%, 100% (defined as the highest jump height), and 130% Acute bicycle challenge for 15 minutes	↔ CK ↓ lactate, NH_3_, knee injury index ↑ contact time drop jumps (muscle strength)	↓ ALT, ALP, TG ↑ HDL-c ↔ AST, LDH, albumin, glucose

↑: statistically significant increase; ↔: change without statistical significance; ↓: statistically significant decrease; M: men; W: women; CK: creatine kinase; IL-6: interleukin-6; IL-8: interleukin-8; IL-10: interleukin-10; TNF-*α*: tumor necrosis factor-alpha; MVC torque: maximum voluntary contraction torque; ROM: range of motion; ALT: alanine aminotransferase; ALP: alkaline phosphatase; AST: aspartate aminotransferase; TG: triglycerides; HDL-c: high-density lipoprotein; LDH: lactate dehydrogenase; ADL: activities of daily living; T2: transverse relaxation time; d-ROMs: derivatives of reactive oxygen metabolites; BAP: biological antioxidant potential; Exp: experiment; PRE: PRE group; POST: POST group; CON: control group; NCE: nanobubble water curcumin extract; RM: repetition maximum.

**Table 3:** Effects of different concentrations and exposure time of curcumin before and/or after intense physical exercise on different markers.

Evaluated markers	>200 mg of curcumin/day		200–300 mg of curcumin/day		≥300 mg of curcumin/day
	≤7 days		≤7 days	>7 days to 28 days	≤7 days
	BE	AE	BE+AE	AE	BE+AE
Proinflammatory cytokines	*n* = 1	*n* = 1	*n* = 1	—	*n* = 1
Anti-inflammatory cytokines	—	—	—	—	*n* = 1
CK	*n* = 2	*n* = 2	*n* = 1	*n* = 1	*n* = 1
Muscle pain	*n* = 2	*n* = 2	*n* = 1	—	*n* = 1
Muscle performance	*n* = 2	*n* = 2	*n* = 1	*n* = 1	—
Lactate, NH_3_	—	—	—	*n* = 1	—
Biochemical (ALT, ALP, TG, HDL, glucose)	—	—	—	*n* = 1	—
Oxidative stress	*n* = 1	*n* = 1	—	—	—

BE: before exercise; AE: after exercise; BE+AE: before and after exercise; *n*: number of studies.

## Data Availability

The data can be made available upon request to the email kelly.dias@ufv.br.

## Supplementary Materials

Supplementary Materials Table S1: full search strategy in PubMed, Scopus, Web of Science, and Embase, including search terms and filters.
