# Supplementary material for: Effects of Curcumin Supplementation on Inflammatory Markers, Muscle Damage, and Sports Performance during Acute Physical Exercise in Sedentary Individuals
Source: Oxid Med Cell Longev. 2021 Oct 7;2021:9264639. doi: 10.1155/2021/9264639 (PMC8516555; doi:10.1155/2021/9264639)
Supplement: Supplementary Materials — Table S1: full search strategy in PubMed, Scopus, Web of Science, and Embase, including search terms and filters. [file 9264639.f1.docx]

**Table S1:** Full search strategy in PubMed, Scopus, Web of Science, and Embase, including search terms and filters.

| **Database** | **Descriptors** | **Items Found** | **Time** | **Date** |
| --- | --- | --- | --- | --- |
| **P**  **u**  **b**  **M**  **e**  **d** | Curcumin: #1  ("curcumin"[MeSH Terms] OR curcumin [TIAB] OR "curcuma" [MeSH Terms] OR turmeric [TIAB]) | 19.011 | 07:47 | 28/06/21 |
|  | Exercise: #2  ("exercise" [MeSH Terms] OR exercise [TIAB] OR resistance [TIAB] OR acute exercise [TIAB] OR aerobic exercise [TIAB] OR "sports" [MeSH Terms] OR sports [TIAB]) | 1.257.560 | 07:52 | 28/06/21 |
|  | Human: #3  ("Human" [MeSH Terms] OR Human [TIAB]) | 2.628.691 | 07:54 | 28/06/21 |
|  | **Total: #1 and #2 and #3** | 330 | 08:00 | 28/06/21 |
| **Database** | **Descriptors** | **Items Found** | **Time** | **Date** |
| **S**  **c**  **o**  **P**  **u**  **s** | Curcumin: #1  (TITLE-ABS-KEY (curcumin) OR TITLE-ABS-KEY(curcuma) OR TITLE-ABS-KEY(turmeric)) | 38.817 | 12:55 | 28/06/21 |
|  | Exercise: #2  (TITLE-ABS-KEY(exercise) OR TITLE-ABS-KEY (resistance) OR TITLE-ABS-KEY ("acute exercise") OR TITLE-ABS-KEY("aerobic exercise") OR TITLE-ABS-KEY(sports)) | 3.053.579 | 12:58 | 28/06/21 |
|  | Human: #3  (TITLE-ABS-KEY(human)) | 23.246.898 | 13:02 | 28/06/21 |
|  | **Total: #1 and #2 and #3** | 1.347 | 13:06 | 28/06/21 |
| **Database** | **Descriptors** | **Items Found** | **Time** | **Date** |
| **W**  **e**  **b**  **O**  **f**  **S**  **c**  **i**  **e**  **n**  **c**  **e** | Curcumin: #1  TS=(curcumin OR curcuma OR turmeric) | 30.152 | 11:50 | 28/06/21 |
|  | Exercise: #2  TS=(exercise OR resistance OR acute exercise OR aerobic exercise OR sports) | 2.325.005 | 11:53 | 28/06/21 |
|  | Human: #3  TS=(Human) | 4.180.318 | 11:56 | 28/06/21 |
|  | **Total: #1 and #2 and #3** | 704 | 12:00 | 28/06/21 |
| **Database** | **Descriptors** | **Items Found** | **Time** | **Date** |
| **E**  **M**  **B**  **A**  **S**  **E** | Curcumin: #1  (curcumin OR curcuma OR 'curcuma longa' OR turmeric) | 35.053 | 11:32 | 28/06/21 |
|  | Exercise: #2  (exercise OR resistance OR 'acute exercise' OR 'aerobic exercise' OR sport OR 'exercise test') | 1.987.743 | 11:36 | 28/06/21 |
|  | Human: #3  (human) | 24.557.631 | 11:40 | 28/06/21 |
|  | **Total: #1 and #2 and #3** | 2.950 | 11:44 | 28/06/21 |
